# Fabrication, characterization and adsorption properties of cucurbit[7]uril-functionalized polycaprolactone electrospun nanofibrous membranes

**DOI:** 10.3762/bjoc.15.97

**Published:** 2019-04-29

**Authors:** Changzhong Chen, Fengbo Liu, Xiongzhi Zhang, Zhiyong Zhao, Simin Liu

**Affiliations:** 1 The State Key Laboratory of Refractories and Metallurgy, School of Chemistry and Chemical Engineering, Wuhan University of Science and Technology, Wuhan 430081, China; 2 School of Chemistry, Biology and Environmental Engineering, Xiangnan University, Chenzhou 423000, China

**Keywords:** adsorption, cucurbit[7]uril, electrospinning, macrocycles, methylene blue, nanofiber

## Abstract

The fabrication of electrospun nanofibers comprising cucurbit[7]uril (CB[7]) and poly(ε-caprolactone) (PCL) is reported. Various techniques such as SEM, FTIR, XRD, DSC and TG were utilized to characterize the morphology, composition and properties of the nanofibers. Uniform bead-free electrospun nanofibers were obtained from PCL/CB[7] mixed solutions and the average fiber diameter of the nanofibers increases with the increase of CB[7] content. The nanofibers are composed of a physical mixture of PCL and CB[7], and CB[7] itself is present in the PCL fiber matrix in an uncomplexed state. The static adsorption behavior of the PCL/CB[7] nanofibers towards methylene blue (MB) was also preliminary investigated. The results indicate that the adsorption of MB onto the nanofibrous membranes fits the second-order kinetic model and Langmuir isotherm model.

## Introduction

Electrospinning is recognized as the most simple and versatile method to fabricate multifunctional nanofibers from the solution or melt of polymers, polymer/nanoparticle mixtures or some low-molecular weight organic compounds [1–5]. With the unique advantages of ultrafine diameter, high specific surface area, controllable morphology, and easy functionalization, electrospun nanofibers have potential applications in various areas including drug delivery and tissue engineering [6], energy storage [7], biosensors [8], catalysis [9], and environmental engineering [10].

Various supramolecular host molecules such as cyclodextrins (CDs), calix[*n*]arenes, and pillar[*n*]arenes can form host–guest inclusion complexes (ICs) with numerous compounds due to their unique cavity present in the molecular structure. Combined with merits of host molecules and electrospun nanofibers, the supramolecular host functionalized nanofibers have been widely reported in recent years as efficient molecular filters and absorbent for the removal of hazardous chemicals or polluting substances. A series of CD-functionalized electrospun nanofibers in the forms of CD-pseudopolyrotaxane [11–12], CDs/polymer [13–15], CD-ICs/polymer [16–17], and polymer-free CDs [5,18] or CD-ICs [19] have been fabricated. In addition, calix[8]arene (C[8])/polyacrylonitrile (PAN) composite nanofiber membranes [20] and supramolecular polymer nanofibers based on pillar[5]arene [21] also were prepared by the electrospinning technique.

**Scheme 1:**
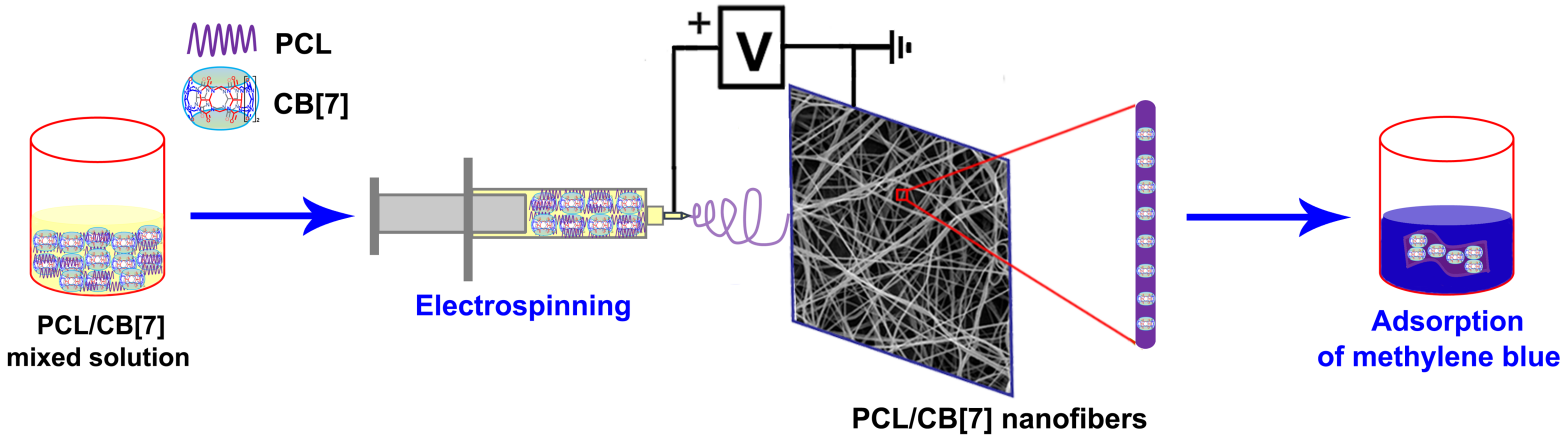
Schematic illustration of the fabricating process of PCL/CB[7] composite nanofibers and the adsorption of methylene blue.

Cucurbit[*n*]urils (CB[*n*]s, *n* = 5–8, 10) are a family of pumpkin-shaped cyclic host molecules containing a hydrophobic cavity surrounded by two identical hydrophilic portals [22–24]. Through hydrophobic effects, ion–dipole interactions, and/or hydrogen bonding, CB[*n*]s show selective molecular recognition properties towards cationic and neutral guests [25–26]. In the last two decades, CB[*n*]s have been used for a variety of applications including supramolecular catalysis/nanoreactor and supramolecular polymers, etc. [25,27–32]. Unlike other hosts such as CDs, calix[*n*]arenes, or pillar[*n*]arenes, the fabrication of CB[*n*]-functionalized nanofibers by electrospinning is a challenging task due to the poor solubility of CB[*n*] in common solvents. To develop CB[*n*]-containing functional materials, the feasibility of CB[7] (the chemical structure is shown in Supporting Information File 1, Figure S1)-based nanofibers through electrospinning was studied for the first time. In addition, the adsorption properties of the fabricated nanofibers were also preliminarily investigated in this work (Scheme 1).

## Results and Discussion

As a common biodegradable polymer, PCL has been frequently reported as the fiber template material for the fabrication of electrospun nanofibers. Various common solvents such as acetone, dichloromethane, chloroform, tetrahydrofuran, DMF or their combinations were used to prepare PCL electrospinning solutions. In this work, a formic acid solution of CB[*n*] and a DMF solution of PCL were mixed as the electrospinning solution for PCL/CB[*n*] nanofibers. These solvents have been selected due to the fact that CB[*n*] is soluble in formic acid and DMF is a good solvent for PCL. Turbidity/precipitation was observed when the volume ratio of the formic acid solution of CB[*n*] in the mixed solution was higher than 20% due to the poor solubility of PCL in formic acid solution. In order to keep the CB[*n*] content in the mixed solution as large as possible, the ratio of formic acid/DMF was fixed to 20:80 (v/v).

Initially, a series of bead-free PCL/CB[*n*] (*n* = 5, 6, 7, 8) nanofibers with different CB[*n*] loading percentages were fabricated by electrospinning (Scheme 1). The experimental results showed that the solubility of CB[5], CB[6] and CB[8] in formic acid was relatively poor compared with that of CB[7]. The loading percentage range of CB[*n*] in the PCL/CB[*n*] composite nanofibers was between 0–25% (w/w, with respect to PCL, similarly hereinafter) for CB[5], and CB[6] and between 0–5% for CB[8] (due to a very poor solubility of CB[8] in formic acid). The surface morphology of the PCL/CB[5], PCL/CB[6], and PCL/CB[8] composite nanofibers as observed by SEM are supplied in Supporting Information File 1 (Figures S2–S4). In contrast, CB[7] was quite soluble in formic acid and the maximum CB[7] mass percentage in the composite nanofibers could be up to 66.67% (the mass ratio of PCL/CB[7] is 100:200), which is far higher than the CDs content in CD-functionalized nanofibers [13–15] and the calixarene content in the calixarene-functionalized nanofibers [20].

**Figure 1:**
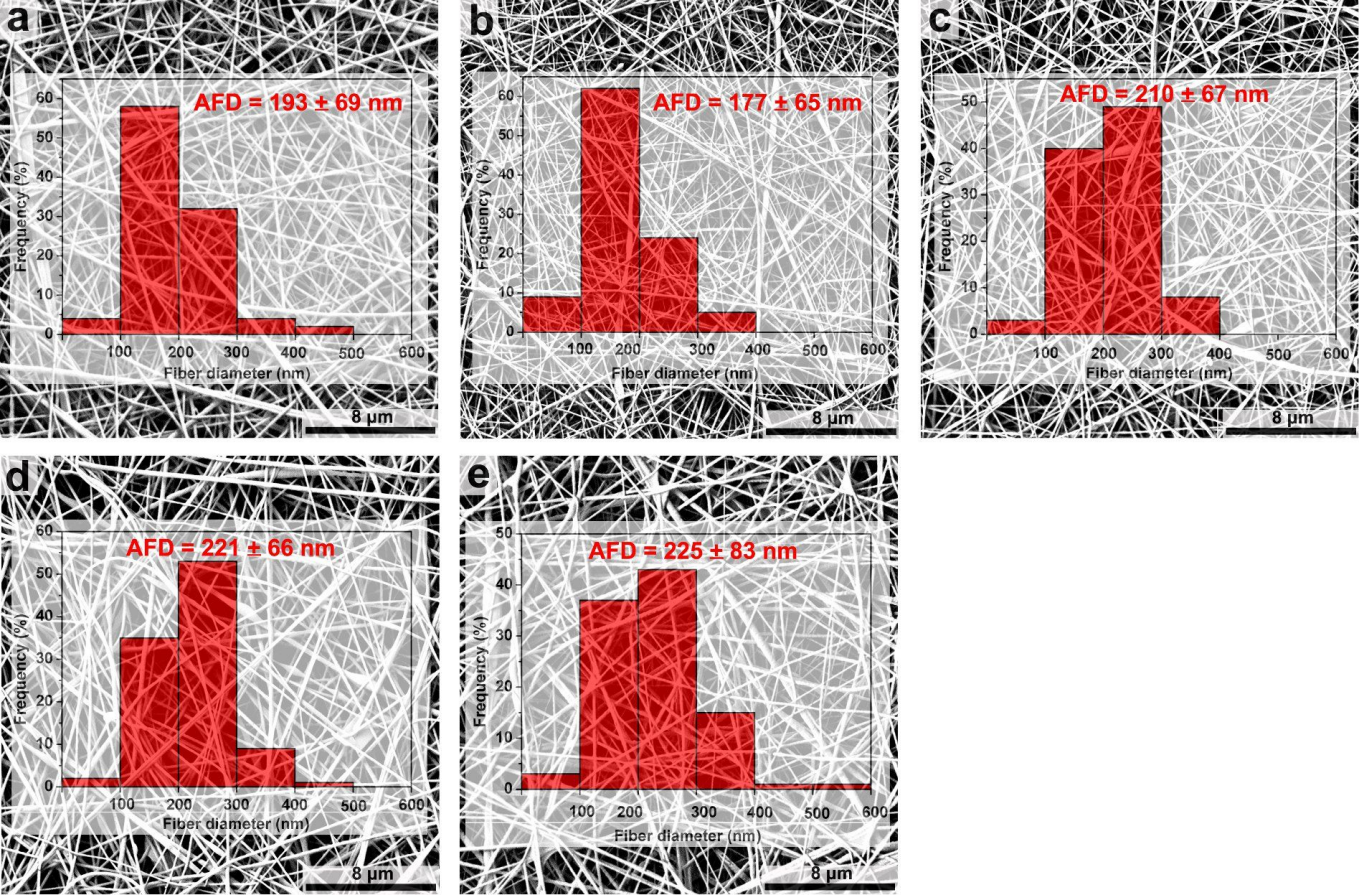
Representative SEM images and the corresponding diameter distribution of the nanofibers: (a) neat PCL; (b) PCL/CB[7] (100:50); (c) PCL/CB[7] (100:100); (d) PCL/CB[7] (100:150); (e) PCL/CB[7] (100:200).

Figure 1 presents the SEM photographs and fiber diameter distributions of neat PCL and the PCL/CB[7] nanofibers with various amounts of CB[7] (the information of compositions, diameter and morphology of electrospun nanofibers are collected in Supporting Information File 1, Table S1). The bead-free neat PCL nanofibers with an average fiber diameter (AFD) of 193 nm were successfully electrospun from the PCL solution in formic acid/DMF with lower concentration (8%, w/v, with respect to the total volume of formic acid and DMF). Furthermore, the diameter of about 57% PCL nanofibers is lower than 200 nm. Compared to the electrospun PCL nanofibers in reported literatures [12,15–17], the AFD of neat PCL nanofibers in this work is obviously smaller, which would bring higher specific surface area of the nanofibers. Thus, the type of solvent in the electrospinning process is a very important factor for the AFD of nanofibers.

It was found that there are no obvious differences in the morphological structures of the PCL/CB[7] (100:50) nanofibers when compared with the neat PCL nanofibers (Figure 1a and 1b). With the increase of CB[7] loading, a few shuttle-shaped beads in the nanofibrous membranes appeared, and the surface of the composite nanofibers became more and more rough or lumpy (Figure 1c–e), suggesting partial phase separation and aggregation of CB[7] on the surfaces. Generally, such imperfect morphologies of electrospun composite nanofibers are expected in the case of two immiscible components, in which the component with lower molecular weight segregates to the surface of the nanowebs. Similar observations have been also reported for CD-functionalized nanofibers [12] and calixarene-functionalized nanofibers with high loading [20].

The diameter distribution and AFD of the PCL/CB[7] nanofibers also varied with the increase of CB[7] loading. When the mass ratio of PCL/CB[7] was 100:50, the diameter of more than 60% of nanofibers was in the range of 100–200 nm, and the corresponding AFD reached the minimum value (only about 177 nm). As the mass ratio of PCL/CB[7] was increased to 100:100, the AFD increased to 210 nm and the nanofibers with the diameter range of 200–300 nm were more than those with the diameter range of 100–200 nm. For samples of PCL/CB[7] (100:150) and PCL/CB[7] (100:200), the AFD was about 221 nm and 225 nm, respectively, and the diameter distributions of the two samples were similar to those of PCL/CB[7] (100:100). These results show that the addition of CB[7] with different loadings influences the morphology and AFD of the resulting electrospun nanofibers. The variations in AFD of the PCL/CB[7] nanofibers are attributed to differences in electrical conductivity and viscosity of the polymer solutions [13–15].

FTIR spectroscopy is a frequently used and effective method to confirm the presence of components of nanowebs and their interactions. According to the FTIR spectra (Figure S5 in Supporting Information File 1), CB[7] is present in the electrospun PCL/CB[7] nanofibers and no chemical reaction occurred between PCL and CB[7] in the nanofibers.

**Figure 2:**
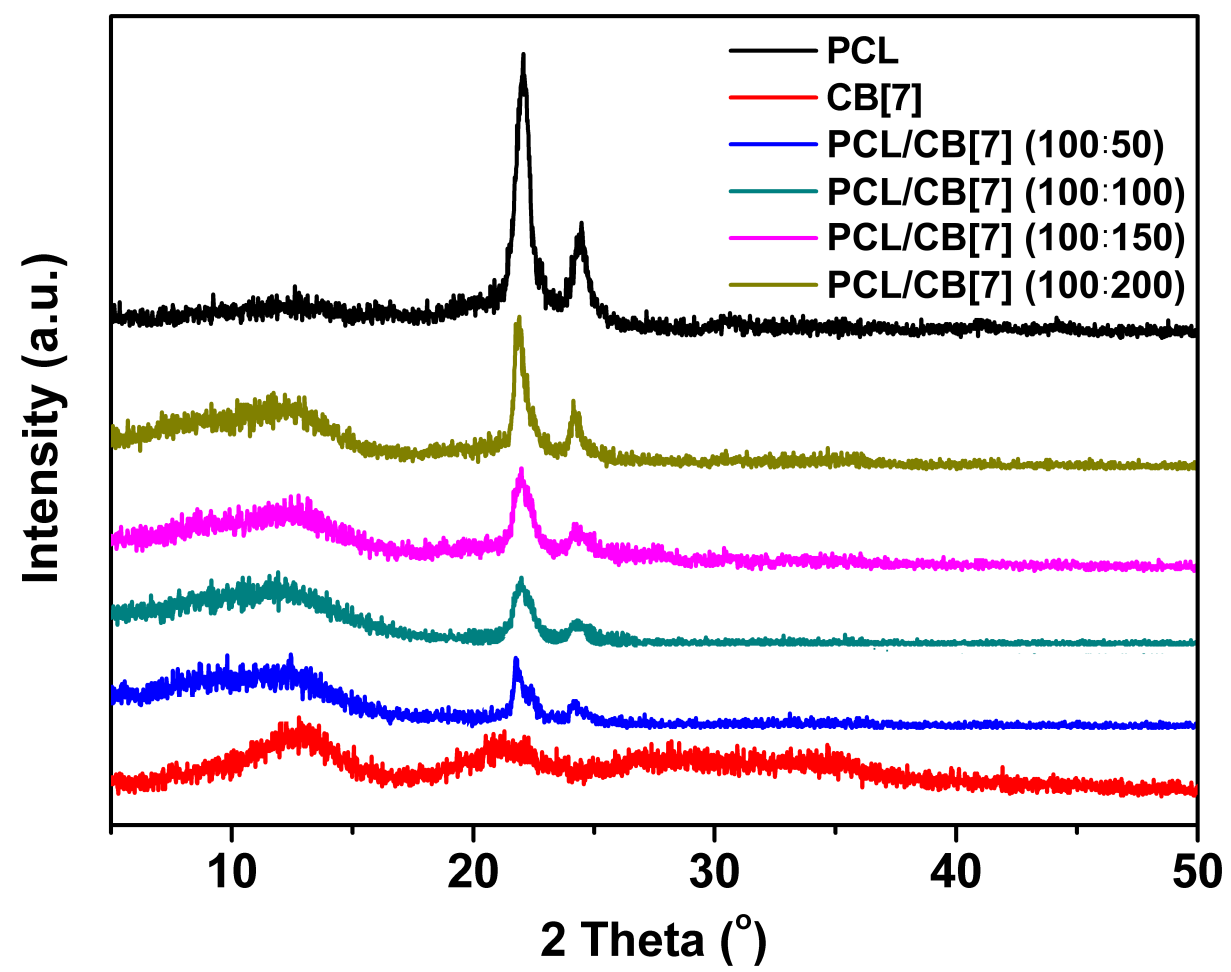
XRD curves of PCL, CB[7] and the PCL/CB[7] nanofibers.

In order to investigate the dispersion and crystallization of CB[7] and PCL in the composite nanofibers, XRD patterns of neat PCL nanofibers and all PCL/CB[7] nanofibers were recorded (Figure 2). PCL is a semi-crystalline polymer, and neat PCL nanofibers elicit two strong and sharp characteristic diffraction peaks at 2θ = 22° and 2θ = 24.5° which correspond to (110) and (200) reflections, respectively [12,15,17]. On the other hand, there are two wider diffraction peaks at 2θ = 13° and 2θ = 21.2° in the XRD curve of CB[7] powders.

From the XRD curves of the PCL/CB[7] nanofibers, the sharp diffraction peaks of PCL and wide diffraction peak of CB[7] at 2θ = 13° also exist, whereas the diffraction peak at 2θ = 21.2° of CB[7] is merged in the diffraction peak of PCL at 2θ = 22°. Namely, the PCL/CB[7] nanofibers with different mass ratios exhibit superimposed diffractograms of their constituents, i.e., PCL and CB[7]. Compared with neat PCL nanofibers and CB[7] powders, the peak position in all XRD curves of PCL/CB[7] nanofibers shows no shift and no new diffraction peak appears. Thus, it is concluded that the electrospun nanofibers are composed of a physical mixture of PCL and CB[7], and CB[7] is only present on the surface or within the PCL/CB[7] nanofibers without forming PCL/CB[7] ICs or PCL/CB[7] pseudorotaxane.

Another interesting observation is that the intensity of all the diffraction peaks for PCL/CB[7] nanofibers increases with the increase of CB[7] content. The phenomenon may be caused by a variation of the continuous phase and crystal aggregates of PCL and CB[7]. At low CB[7] contents, the majority of the CB[7] molecules could disperse homogeneously in the PCL matrix, partly causing the disruption of continuous phase and crystallization of PCL and CB[7]. With the increase of CB[7] content, there are more continuous phases of CB[7] and PCL formed in the PCL/CB[7] nanofibers, which causes the formation of more crystal aggregates.

**Figure 3:**
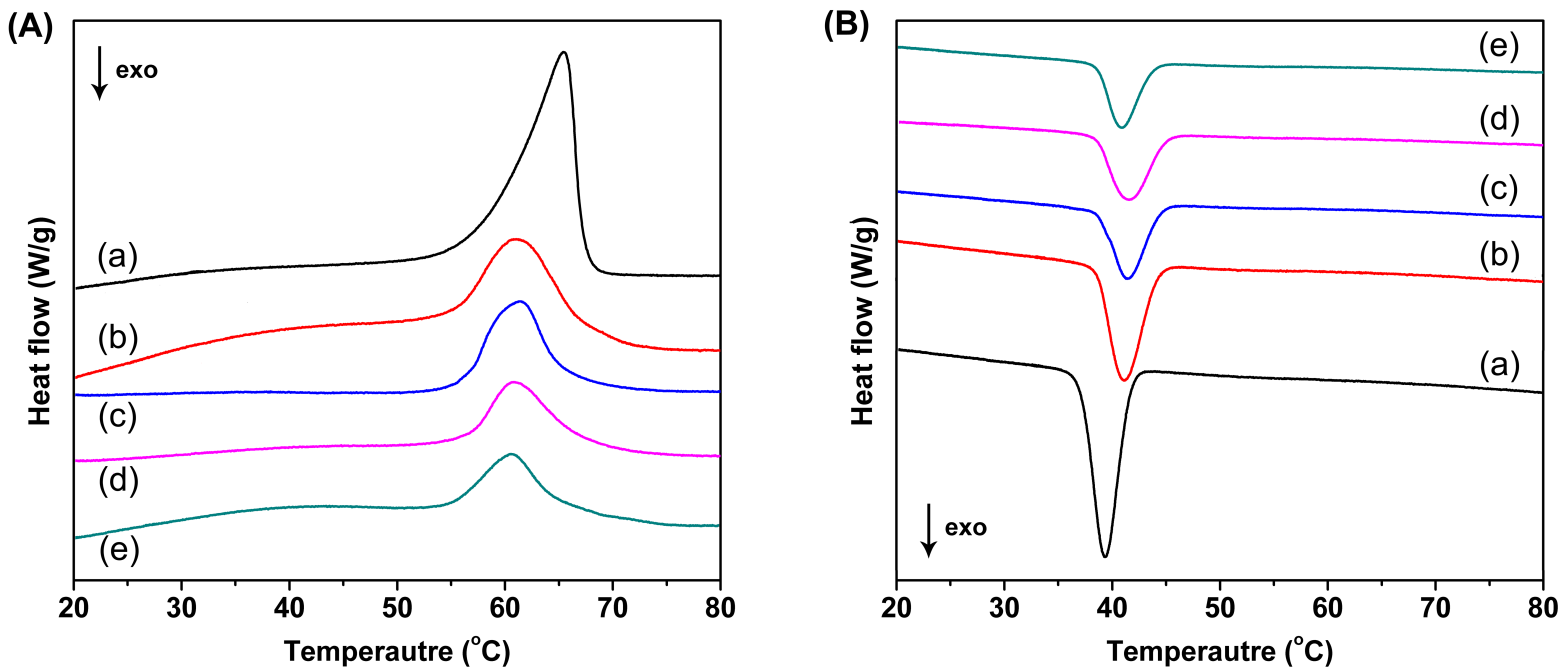
DSC thermograms of nanofibers for the melting cycle (A) and cooling cycle (B). (a) neat PCL; (b) PCL/CB[7] (100:50); (c) PCL/CB[7] (100:100); (d) PCL/CB[7] (100:150); (e) PCL/CB[7] (100:200).

DSC measurements can provide valuable information about the thermal transition properties, i.e., phase-transition temperatures and transition enthalpies. Figure 3 shows the first heating and cooling thermograms of neat PCL and electrospun PCL/CB[7] nanofibers. For comparison, the DSC curves of neat CB [7] and PCL are also provided (Figure S6 in Supporting Information File 1). Neat CB [7] has no strong and sharp peaks below 100 °C, indicating that there is no phase transition at that temperature. There is only a very wide and weak peak in the heating cycle, which was caused by evaporating of water from the cavity of CB[7] (Figure S7 in Supporting Information File 1).

As can be deduced from Figure 3, all electrospun webs exhibit obvious melting and crystallization peaks below 100 °C, though their peak intensity seem to be different from each other. The melting/crystallization temperatures, melting/crystallization enthalpies, and calculated PCL crystallinities of the nanofibrous membranes are listed in Table S2 (Supporting Information File 1). It is found that the melting temperature (*T*_m_) of neat PCL is about 65.4 °C, and the *T*_m_ of the electrospun PCL/CB[7] nanofibers with different CB[7] loadings decreased by 4–5 °C. On the other hand, the crystallization temperature (*T*_c_) of neat PCL is 39.3 °C, while the electrospun PCL/CB[7] nanofibers exhibit crystallization at elevated temperatures (at around 41 °C).

Tonelli et al. reported PCL/PCL-α-CD-IC nanofibers [17] and PCL/α-CD pseudorotaxane nanofibers [12] obtained by electrospinning. They found that the variations of phase-transition temperatures of nanofibers compared with those of neat PCL a) both, *T*_m_ and *T*_c_ of PCL/PCL-α-CD-IC nanofibers increased, b) *T*_m_ of PCL/α-CD pseudorotaxane nanofibers was almost the same and their *T*_c_ increased, and c) *T*_m_ of uncomplexed PCL/40% α-CD composite nanofibers decreased and their *T*_c_ increased. Apparently, the change tendency of *T*_m_ and *T*_c_ of the PCL/CB[7] nanofibers reported in this work matches those of PCL/40% α-CD composite nanofibers, which further confirms that CB[7] is present in the PCL fiber matrix in an uncomplexed state. In other words, the PCL/CB[7] nanofibers are just the physical mixture of the two components, and the PCL chains haven’t occupied the cavities of CB[7]. It was expected that CB[7] molecules in the nanofibers have free cavities, able to an effective capture of target molecules from the surroundings through the formation of host–guest complexes.

As collected in Table S2 (Supporting Information File 1), the melting enthalpy and crystallization enthalpy of neat PCL nanofibers are 63.57 J/g and 47.09 J/g, respectively. In the case of PCL/CB[7] nanofibers, the enthalpies decreased obviously for both heating and cooling process. The degree of crystallinity of PCL chains can be calculated from the melting enthalpy from DSC curves using Equation 1.

[1] $$\chi_{c}(\%)=\frac{\Delta H_{m}}{\Delta H_{m}^{0}}$$

where *χ_c_* is the degree of crystallinity of the samples, Δ*H_m_* is the experimental melting enthalpy of PCL chains and 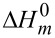 is the theoretical melting enthalpy of a reference PCL with 100% crystallinity. Herein, 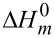 is taken as 135.6 J/g [12,15].

As shown in Table S2 (Supporting Information File 1), the degree of crystallinity of neat electrospun PCL nanofibers is 46.9%, roughly as high as that of PCL nanofibers reported in the literature [17]. Due to the doped PCL chains in the nanofibers, the PCL/CB[7] nanofibers show a lower crystallinity compared with that of neat PCL nanofibers. The degree of crystallinity (*χ*_c_) gradually increases from PCL/CB[7] (100:50) to PCL/CB[7] (100:200), indicating the more perfect crystallization of PCL chains in the nanofibers with the increase of CB[7] content. This result agrees with the observation from the XRD curves.

The TG and DTG curves of neat PCL, CB[7] and electrospun PCL/CB[7] nanofibers in the temperature range of 25–650 °C are depicted in Figure S7 (Supporting Information File 1). From the TG/DTG curves, the thermal decomposition temperatures of the PCL/CB[7] nanofibers are higher than that of CB[7] alone, demonstrating a better thermal stability of the PCL/CB[7] nanofiber composites.

Methylene blue (MB) is frequently used as a routine model dye molecule to test the adsorption capability of materials. More importantly, the binding constant of MB with CB[7] is as high as 10^7^ M^−1^ in aqueous solution [33]. However, due to the considerable solubility of CB[7] in water, the adsorption experiment in this work was carried out in ethanol instead of water.

**Figure 4:**
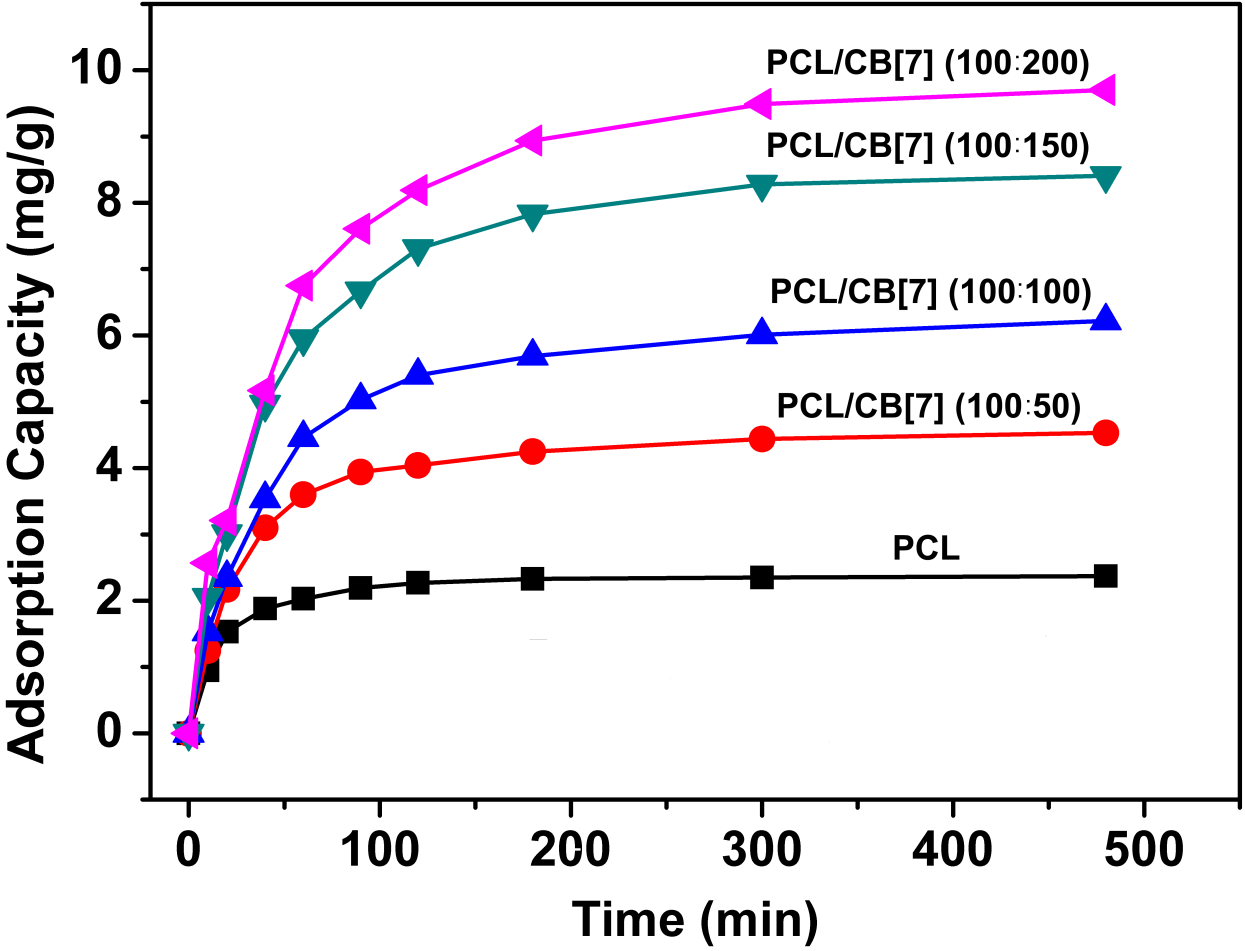
Adsorption kinetics curve of the adsorption of methylene blue (MB) by the electrospun nanofibrous membranes.

The adsorption capacity of MB by PCL and the PCL/CB[7] nanofiber membranes as a function of time are shown in Figure 4. Neat PCL nanofibrous membranes shows very low adsorption capability for MB, and the corresponding *q*_e_ is only about 2 mg·g^−1^. Compared to the neat PCL nanofibrous membrane, all the PCL/CB[7] nanofibrous membranes exhibit increased adsorption capability for MB, indicating that the incorporation of CB[7] plays a positive role in the promotion of adsorption capability. Meanwhile, there is an obvious tendency that the equilibrium adsorption capability of PCL/CB[7] nanofibrous membranes increased with the increase of CB[7] content in the nanofibers.

Compared with the reported works on CB[*n*]-based absorbents [34–36], the maximum adsorption capacity of MB by PCL/CB[7] nanofibrous membranes is relatively low. The unexpected low adsorption capacity for MB should be mainly ascribed to the weak binding between CB[7] and MB in ethanol, as in non-aqueous solvents the hydrophobic effect is tremendously weakened. Another possible reason is that the portals of CB[7] are blocked by the PCL chains, which doesn’t allow MB to enter the cavity of CB[7] even when the cavity is empty according to above DSC results.

Various adsorption kinetics models were used to explain the adsorption mechanism and to determine the intrinsic adsorption kinetic constant. Usually, the pseudo-first-order model, pseudo-second-order model and intraparticle diffusion model are used to evaluate adsorption kinetics behavior (the equations of the three models are given in Supporting Information File 1). According to the experimental data, the adsorption kinetic data of PCL and PCL/CB[7] nanofibrous membranes fitting by the above three adsorption kinetics models are listed in Table S3 (Supporting Information File 1). As can be seen the correlation coefficients (*R*^2^) of all nanofibrous membranes in the pseudo-second-order kinetic model are higher than those of the pseudo-first-order kinetic model and the intraparticle diffusion model, indicating that the adsorption of MB onto electrospun nanofibrous membranes fits a pseudo-second-order kinetic.

The relationships between *t*/*q*_t_ and *t* for all the PCL/CB[7] nanofibrous membranes are shown in Figure S8 (Supporting Information File 1), which meet a good linear relationship by the linear simulation and further confirm the pseudo-second-order kinetic of PCL/CB[7] nanofibrous membranes. Actually, the adsorption kinetics of the PCL/CB[7] nanofibrous membranes agree with those of calixarene-functionalized [20] and CD-functionalized [37] nanofibrous membranes. The adsorption isotherm of MB by nanofibrous membranes at 293 K was investigated, and PCL/CB[7] (100:100) nanofibrous membrane was selected as the typical research object here. There are two well-known adsorption isotherm models to examine the equilibrium isotherm of adsorbates by adsorbents: the Langmuir isotherm model and Freundlich isotherm model (the equations are given in Supporting Information File 1).

**Figure 5:**
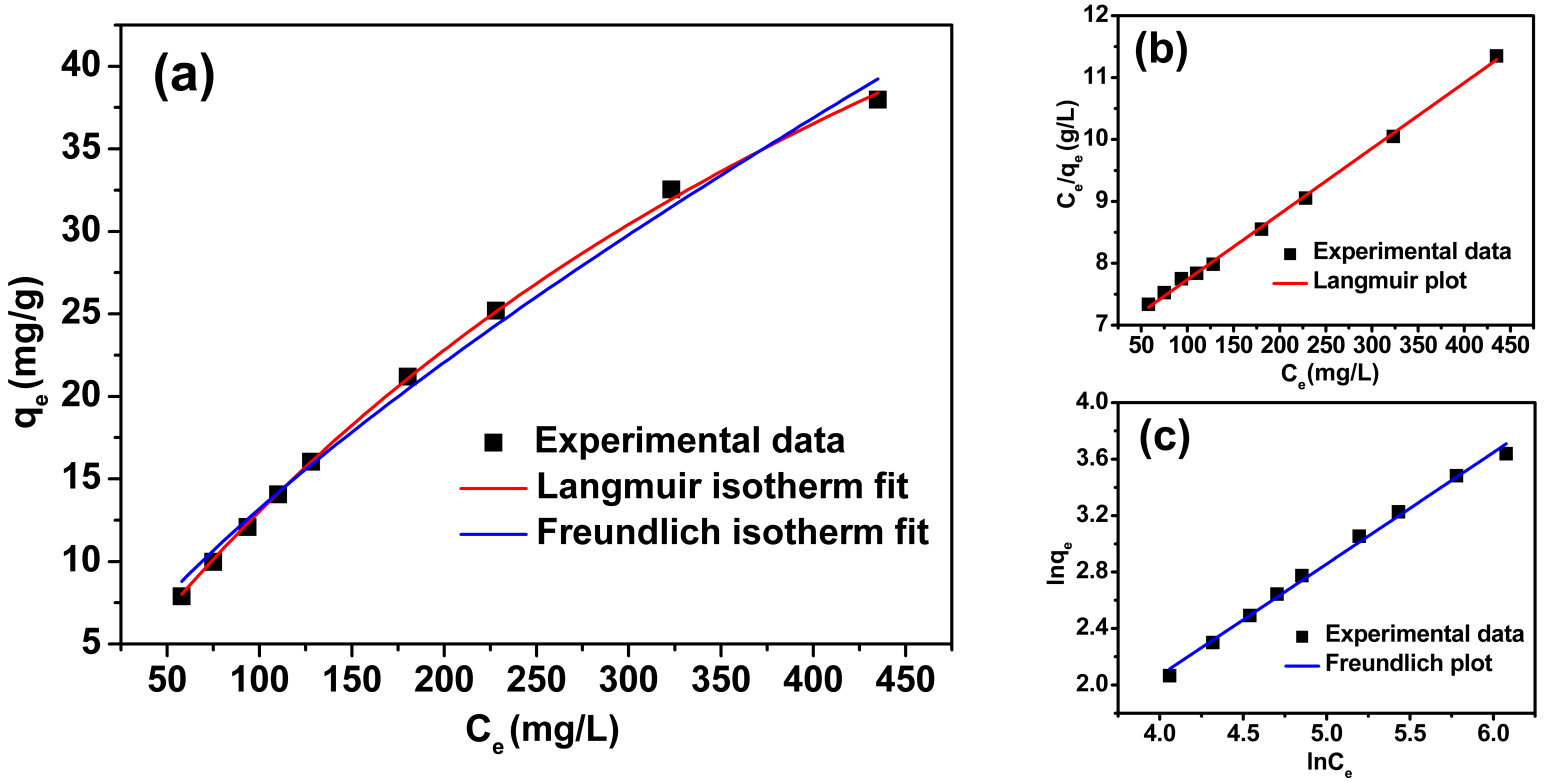
Adsorption isotherms (a) and the corresponding Langmuir plot (b) and Freundlich plot (c) for MB adsorption onto the electrospun nanofibrous membranes.

Figure 5a shows the isotherms of the above two models, and Figure 5b and 5c depict the corresponding linear fitting plot of the Langmuir and Freundlich isotherm model, respectively. The related equilibrium parameters obtained from Figure 5b and 5c are summarized in Table 1. According to the comparison of correlation coefficients *R*^2^, it is apparent that the experimental equilibrium adsorption data of MB by PCL/CB[7] nanofibrous membranes are better fitted with the Langmuir isotherm model. Therefore, it is easily concluded that the adsorption of MB took place at specific homogeneous sites within the nanofibrous membrane and formed a monolayer coverage of MB at the surface of the nanofibrous membrane [34].

**Table 1:** Langmuir and Freundlich isotherm fitting parameters for the adsorption of MB onto PCL/CB[7] (100:100) nanofiber membranes.

Sample	Langmuir isotherm				Freundlich isotherm		
			
	*q*_max_ (mg·g^−1^)	*b* (L·mg^−1^)	*R*^2^		*K*_F_	*n*	*R*^2^

PCL/CB[7] (100:100)	91.659	0.07187	0.9992		0.43432	1.3489	0.9926

The morphology of PCL and PCL/CB[7] nanofibers after the absorption experiment are shown in Figure S9 (Supporting Information File 1). It is found that all nanofibers kept their fibrous shape after the absorption experiment, which indicates that the nanofibrous membranes have good mechanical properties and can be easily removed from the adsorption tail liquid.

## Conclusion

CB[7]-based nanofibers with varying CB[7] contents were prepared by electrospinning from PCL/CB[7] mixed solutions. SEM results show that bead-free nanofibers with uniform diameter distribution were obtained and the maximum CB[7] mass percentage in the composite nanofibers can reach to 66.67% (the mass ratio of PCL/CB[7] is 100:200). XRD patterns and DSC curves confirmed that PCL and CB[7] in the electrospun nanofibers are present as a physical mixture, and the cavities of the CB[7] molecules are not occupied by the PCL chains. It is found that the adsorption kinetics of MB by PCL/CB nanofibrous membranes fitted a second-order kinetic model, and the corresponding adsorption isotherm fitted the Langmuir isotherm model. Although the adsorption capability of the nanofibrous membranes was low due to weak binding between CB[7] and the dye molecule, it was greatly improved with the addition of CB[7]. Currently the fabrication of CB[*n*]s linked covalently on rigid polymeric matrices through the electrospinning technique is on the way, in order to find a real application on the removal of organic wastes in water.

## Supporting Information

File 1 Experimental data, additional tables and images.
