# Supplementary material for: Fabrication, characterization and adsorption properties of cucurbit[7]uril-functionalized polycaprolactone electrospun nanofibrous membranes
Source: Beilstein J Org Chem. 2019 Apr 29;15:992–9. doi: 10.3762/bjoc.15.97 (PMC6541341; doi:10.3762/bjoc.15.97)
Supplement: File 1 — Experimental data, additional tables and images. [file Beilstein_J_Org_Chem-15-992-s001.pdf]

BEILSTEIN JOURNAL OF ORGANIC CHEMISTRY

Supporting Information

for

Fabrication, characterization and adsorption properties of cucurbit[7]uril-functionalized polycaprolactone electrospun nanofibrous membranes

Changzhong Chen, Fengbo Liu, Xiongzhi Zhang, Zhiyong Zhao and Simin Liu

Beilstein J. Org. Chem. **2019,** *15,* 992–999. doi:10.3762/bjoc.15.97

Experimental data, additional tables and images

License and Terms: This is a supporting information file under the terms of the Creative Commons Attribution License (http://creativecommons.org/licenses/by/4.0). Please note that the reuse, redistribution and reproduction in particular requires that the authors and source are credited.
The license is subject to the *Beilstein Journal of Organic Chemistry* terms and conditions: (https://www.beilstein-journals.org/bjoc)

1. Experimental part

Materials

Polycaprolactone (PCL) with viscosity-average molecular weight of 80,000 was purchased from Shenzhen Esun Industrial Co., Ltd., China. Cucurbit[7]uril (CB[7], as shown in Figure S1) was synthesized by our lab according to the reported literature [1]. Methylene blue (MB, $C_{16}H_{18}ClN_3S{\cdot}3H_2O$) was produced by Tianjin No.1 Chemical Reagent Factory, China. Analytical reagent *N,N*-dimethylformamide (DMF), ethanol and formic acid (88%) were produced by Tianjin Kemiou Chemical Reagent Ltd., China, Tianjin Deen Chemical Reagent Ltd., China, and Tianjin Damao Chemical Reagent Ltd., China, respectively. All reagents were used without further treatment.

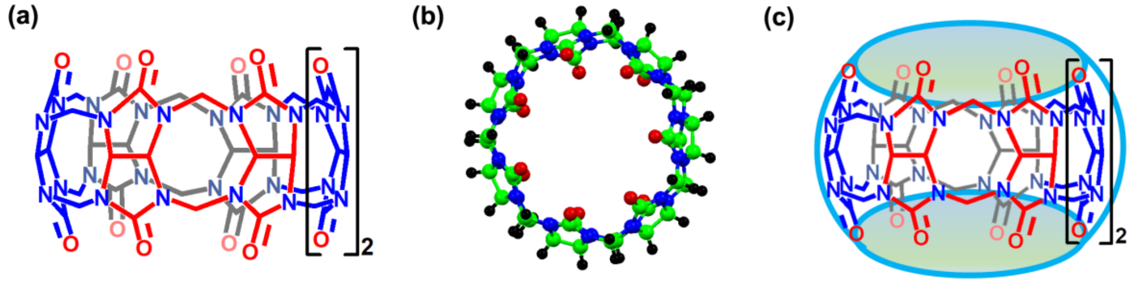

Figure S1: (a) The chemical structure, (b) top view of X-ray crystal structure, and (c) schematic representation of the pumpkin-shaped molecular structure of CB[7].

Electrospinning of PCL/CB[7] composite nanofibers

Firstly, a series of 10% (w/v, with respect to DMF) homogeneous PCL solutions were individually prepared by dissolving PCL in DMF. Also, CB[7] solutions with different concentrations were obtained by dissolving different amounts of CB[7] in formic acid. Then, the PCL solution and CB[7] solution were mixed under stirring for 2 h at the volume ratio of 80:20, and the concentration of PCL decreases to 8% (w/v, with respect to DMF/formic acid). The amount of CB[7] in the mixed solution was 0, 50, 100, 150, or 200 wt % relative to PCL.

The prepared solution for electrospinning was loaded into a 5 mL glass syringe fitted with a metallic needle of 0.7 mm inner diameter. The syringe was fixed horizontally on a single-channel syringe pump (LSP02-1A, Baoding Longer Precision Pump Co. Ltd., China), and the metallic needle tip was connected with the electrode of high voltage power supply (Changzhou Blue-butterfly Automatic Control Fittings Factory, China). According to the optimization of electrospinning parameters, the electrospinning voltage, the solution flow rate, and the tip-to-collector distance were set at 15 kV, 1 mL/h and 15 cm, respectively. The temperature and relative humidity during electrospinning were 25 °C and 30%, respectively.

Characterization

The morphology of the nanofibers was characterized by a Phenom Pro G3 scanning electron microscopy (SEM, Phenom, Holland) operating at 10 kV. Before SEM observation, all samples were thinly sputter-coated with gold. The average fiber diameter (AFD) of the electrospun nanofibers was measured by using an UTHSCSA Image Tool Program from at least 500 fibers in five SEM images for each sample.

Fourier transformed infrared (FT-IR) spectra were measured by a Nicolet 760 FT-IR spectrometer (Nicolet Co., USA) between the wavelengths 4000–400 cm^{-1}. Every sample was blended with KBr powder and then pressed into a small disk for testing.

Both differential scanning calorimetry (DSC) and thermogravimetry (TG) curves of the electrospun fibrous membranes were recorded by a Simultaneous Thermal Analysis Apparatus (STA 449 F3 Jupiter®, Netzsch, Germany) under N_2 atmosphere. For DSC measurements, the samples underwent a heating–cooling cycle between 20 and 100 °C with a scanning rate of 2 °C/min. The TG curves were performed from room temperature to 650 °C at a heating rate of 10 °C/min.

The X-ray powder diffraction (XRD) patterns were recorded with a wide-angle X-ray powder diffractometer (D8 Advance, Bruker-AXS, Germany) in the range of $5° < 2\theta < 50°$ with a collection time of 0.1 s per step.

Adsorption experiment with MB

Batch adsorption experiments of MB onto the nanofibers membranes were performed on a shaker (ZHWY-200B, Shanghai Hasuc Instrument Manufacture Co., Ltd., China) with a shaking speed of 160 rpm. The kinetic experiments were carried out by immersing 10 mg nanofibrous membrane into 50 mL MB solution in ethanol with the initial concentration of 40 mg·L^{-1} at 20 ± 1 °C.

The concentration of MB in the testing solution at different adsorption times was determined by UV–vis spectroscopy (Lambda 650S, PerkinElmer, USA) based on the standard curve. The measurement wavenumber of MB is 655 nm. The adsorption capacity (q, mg·g^{-1}) of MB for each sample was calculated using the following equation:

$$q = \frac{(C_0 - C_e)V}{\omega} \qquad \text{(S1)}$$

where C_0 is the initial concentration of MB in the testing solution (mg·L^{-1}), C_e is the equilibrium concentration of MB in the testing solution (mg·L^{-1}), V is the volume of the testing solution (L), and ω is the mass of the testing nanofibrous membrane (g). Adsorption isotherms at 293 K were conducted with initial concentrations ranging from 60 to 500 mg·L^{-1}.

2. The SEM images of CB[5], CB[6] and CB[8]-based electrospun nanofibers

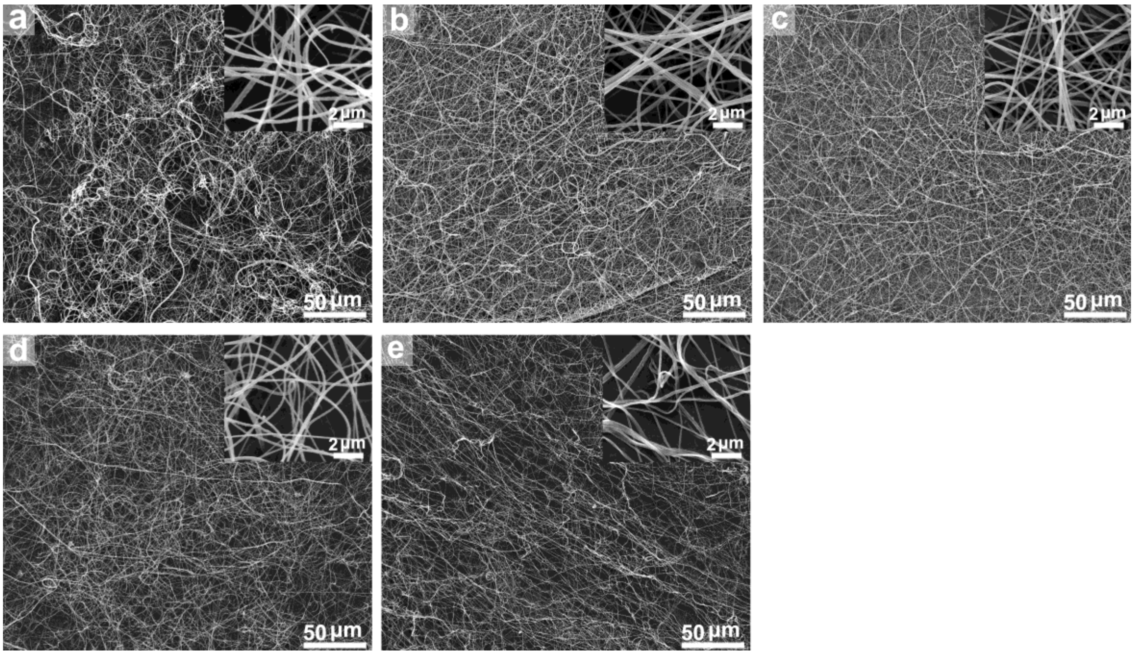

Figure S2: SEM images of PCL/CB[5] nanofibers with different PCL/CB[5] mass ratios: (a) 100:5; (b) 100:10; (c) 100:15; (d) 100:20; (e) 100:25.

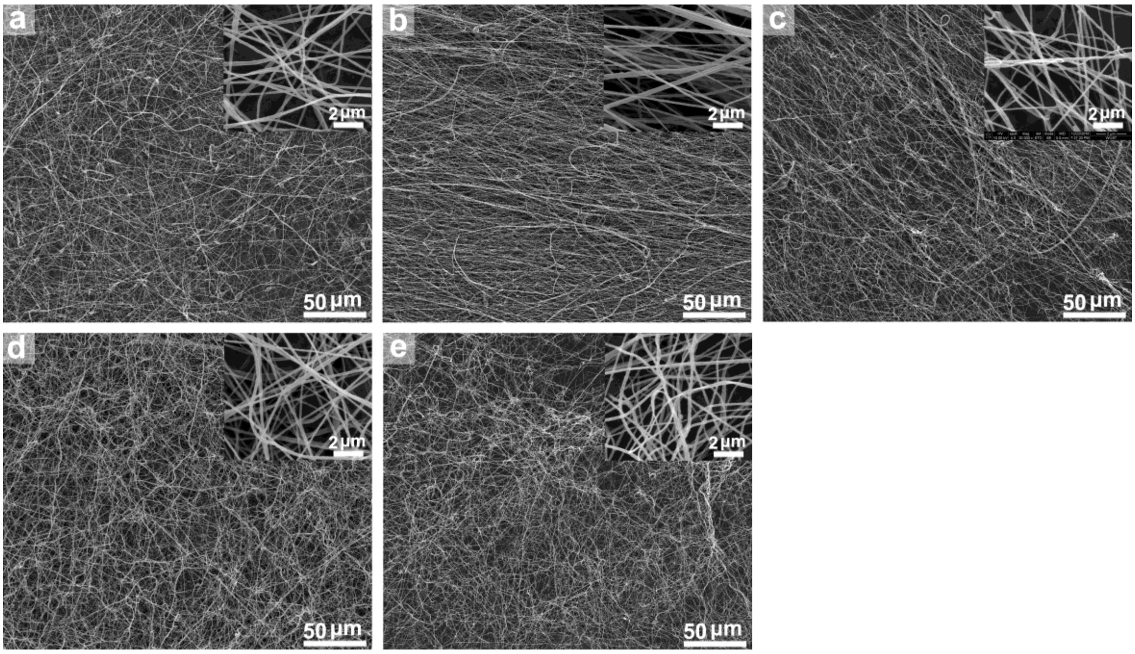

Figure S3: SEM images of PCL/CB[6] nanofibers with different PCL/CB[6] mass ratios: (a) 100:5; (b) 100:10; (c) 100:15; (d) 100:20; (e) 100:25.

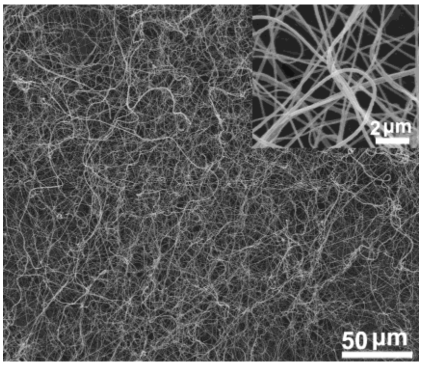

Figure S4: SEM images of PCL/CB[8] nanofibers with the PCL/CB[8] mass ratio of 100:5.

3. The information of compositions, diameter and morphology of electrospun neat PCL and PCL/CB[7] nanofibers

Table S1: Compositions, diameter and morphology of electrospun nanofibers

Sample	Weight percent of PCL (w/v, %)[a]	Weight percent of CB[*7*] (wt%) [b]	Fiber diameter (nm)	Fiber morphology
PCL	8	0	193 ± 69	Beaded-free nanofibers
PCL/CB[7] (100:50)	8	50	177 ± 65	Beaded-free nanofibers
PCL/CB[7] (100:100)	8	100	210 ± 67	Beaded-free nanofibers
PCL/CB[7] (100:150)	8	150	221 ± 66	Beaded-free nanofibers
PCL/CB[7] (100:200)	8	200	225 ± 83	Beaded-free nanofibers

[a] With respect to solvent system (DMF/formic acid). [b] With respect to the polymer (PCL).

4. FTIR spectra of neat PCL, CB[7] and PCL/CB[7] nanofibers

In the FTIR spectrum of neat PCL nanofibers (Figure S5a), the characteristic peaks appearing at 2956 cm^{-1}, 2926 cm^{-1} and 2854 cm^{-1} correspond to CH_2 asymmetric and symmetric stretching. The dominant peaks at 1732 cm^{-1} and 1240 cm^{-1} correspond to C=O stretching and C–C stretching vibrations, respectively. The adsorption bands in the IR spectrum of CB[7] powders (Figure S5b) at around 2922 cm^{-1}, 1735 cm^{-1} and 1469 cm^{-1} are assigned to the CH_2 stretching, C=O stretching, and CH_2 bending vibrations, respectively [2]. The adsorption peaks at 1421 cm^{-1} and 1377 cm^{-1} are attributed to the asymmetric and symmetric C−N stretching vibrations of the glycoluril ring of CB[7]. The characteristic peaks at 1325 cm^{-1}, 1230 cm^{-1}and 1185 cm^{-1} of CB[7] are caused by the combination vibration of C−N/C−C stretching, C−N stretching/C−N bending and C−N stretching/C−H bending vibrations, respectively. In addition, the sharp adsorption peaks at 968 cm^{-1} and 802 cm^{-1}, respectively, correspond to the C−C stretching and deformation vibrations of the glycoluril ring for CB[7].

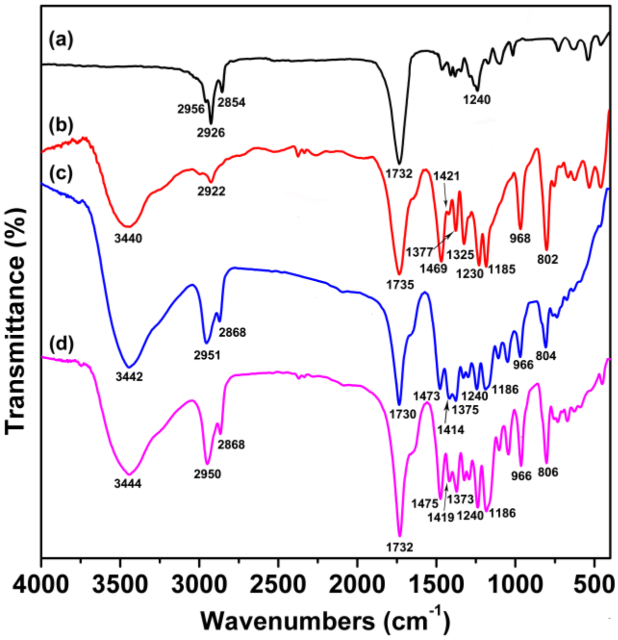

Figure S5: FTIR spectroscopy of (a) PCL, (b) CB[7], (c) PCL/CB[7] (100:100) and (d) PCL/CB[7] (100:200) nanofibers.

In the case of electrospun PCL/CB[7] (100:100) (Figure S5c) and PCL/CB[7] (100:200) nanofibers (Figure S5d), most of salient characteristic adsorption bands shown in the IR spectra of CB[7] and PCL can be observed. Some characteristic bands of groups in PCL/CB[7] (100:100) and PCL/CB[7] (100:200) show minor shifts due to intermolecular interactions (e.g., hydrogen bonding) between PCL and CB[7]. Moreover, no new characteristic peaks are observed in the spectra of PCL/CB[7] (100:100) and PCL/CB[7] (100:200) nanofibers. Therefore, the results confirm that CB[7] is present in the electrospun PCL/CB[7] nanofibers and no chemical reaction occurs between PCL and CB[7] in the nanofibers.

5. DSC thermogram of neat PCL and CB[7]

There are sharp peaks in the heating and cooling process between 20–100 °C for neat PCL, indicating phase transition of PCL in that temperature interval. However, there is only a very wide and weak peak in the heating process during 20–100 °C for neat CB[7], which may be caused by the evaporating of covalent water in cavity of CB[7].

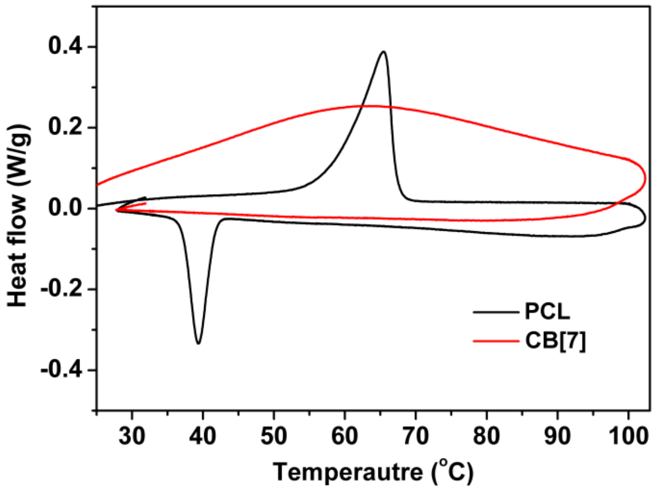

Figure S6: DSC thermogram of neat PCL and CB[7].

6. Thermal characteristics of neat PCL and PCL/CB[7] nanofibers

Table S2: Thermal characteristics of neat PCL and PCL/CB[7] nanofibers.

Sample	T_m (^oC)	T_c (^oC)	$\Delta H_m{}^a$ (J/g)	$\Delta H_m{}^b$ (J/g)	$\Delta H_c{}^c$ (J/g)	$\Delta H_c{}^d$ (J/g)	χ_c (%)
neat PCL	65.4	39.3	63.57	63.57	47.09	47.09	46.9
PCL/CB[7] (100:50)	61.1	41.2	36.93	55.37	32.20	48.28	40.8
PCL/CB[7] (100:100)	61.3	41.1	28.31	56.62	24.51	49.02	41.8
PCL/CB[7] (100:150)	60.4	41.3	23.36	58.40	20.24	50.60	43.1
PCL/CB[7] (100:200)	60.5	41.0	20.25	60.81	17.52	52.61	44.8

Note: [a] Melting enthalpy of the samples. [b] Theoretical melting enthalpy of the PCL chains (mass adjusted). [c] Crystallization enthalpy of the samples. [d] Theoretical crystallization enthalpy of the PCL chains (mass adjusted).

7. TG and DTG analysis

The TG and DTG curves of neat PCL, CB[7] and electrospun PCL/CB[7] nanofibers in the temperature range of 25–650 °C are depicted in Figure S7. From the TG curve of PCL, it is clear that neat PCL has a slight mass loss below 300 °C (<0.65%) and there is only one weight-loss region with the decomposition onset temperature at 386.8 °C. The decomposition peak temperature in the DTG curve of PCL is at about 408.6 °C. In contrast, there are two main weight-loss stages observed from the TG curve of pure CB[7] powders. The first mass-loss stage of CB[7] is in the temperature range of 25–150 °C (the

peak temperature is at 83.6 °C) with about 15.6% mass loss, which resulted from the evaporation of adsorbed small molecules (e.g., water). The result is in accordance with that from the DSC thermogram of neat CB[7] shown in Figure S6. The second mass-loss stage is between 365–510 °C (the peak temperature is at 430.7 °C from Figure S7b) with a weight-loss ratio of 45%, suggesting the decomposition of the CB[7] molecule.

From the TG curves of PCL/CB[7] nanofibers, there are three mass-loss stages corresponding to those of PCL and CB[7]. The decomposition onset temperature of the main mass-loss stage is at 378.8 °C, 377.8 °C, 376.9 °C and 368.5 °C for PCL/CB[7] (100:50), PCL/CB[7] (100:100), PCL/CB[7] (100:150), and PCL/CB[7] (100:200), respectively, which are between the onset temperatures of CB[7] (the second mass loss stage) and PCL. Therefore, the thermal decomposition temperatures of the PCL/CB[7] nanofibers are higher than that for CB[7] alone, demonstrating better thermal stability of the PCL/CB[7] nanofiber composites.

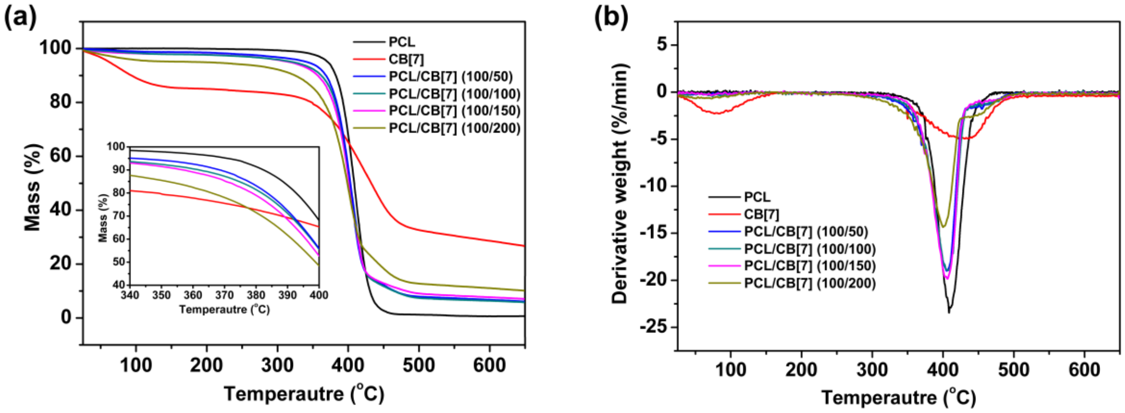

Figure S7: TG curves (a) and DTG curves (b) of PCL, CB[7] and the PCL/CB[7] nanofibers.

8. The equations of pseudo-first-order model, pseudo-second-order model and intraparticle diffusion model

The pseudo-first-order kinetic model described by Lagergren is in a simple linear form, and it follows [3]:

$$\ln(q_e - q_t) = \ln q_e - k_1 t \quad \text{(S2)}$$

where q_e and q_t (mg·g^{-1}) are the adsorption capacity at equilibrium time and time t, respectively, and k_1 (min^{-1}) is the equilibrium rate constant of the pseudo-first-order adsorption.

The pseudo-second-order kinetic model based on the equilibrium adsorption capacity can be expressed as the following equation [3]:

$$\frac{t}{q_t} = \frac{1}{k_2 q_e^2} + \frac{t}{q_e} \quad \text{(S3)}$$

where k_2 (g·mg^{-1}·min^{-1}) is the adsorption rate constant of the pseudo-second-order adsorption.

The intraparticle diffusion model equation described by Weber and Morris is expressed as follows [4]:

$$q_t = k_3 t^{\frac{1}{2}} + C \quad \text{(S4)}$$

where k_3 (min$^{1/2}$·mg·g^{-1}) is the intraparticle diffusion rate constant and C is the intercept.

9. Pseudo-second-order kinetic plots for MB adsorption onto the electrospun nanofibrous membranes

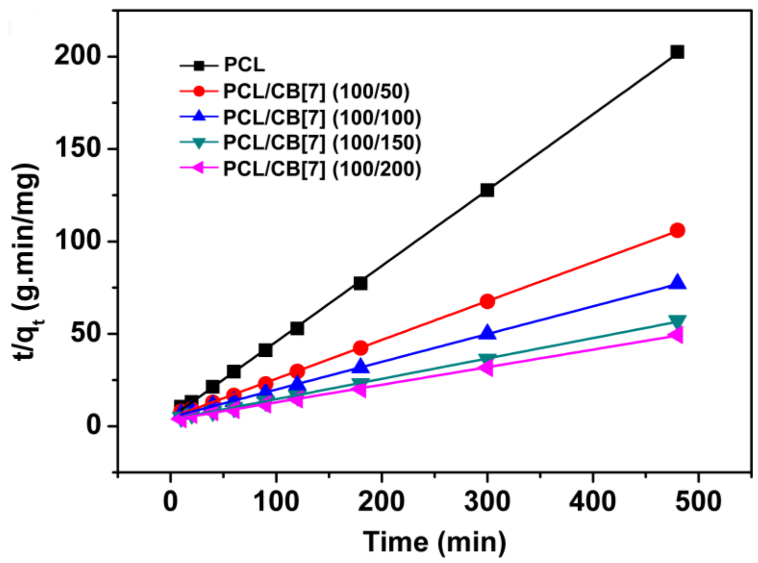

Figure S8: Pseudo-second-order kinetic plots for MB adsorption onto the electrospun nanofibrous membranes.

10. The equations of Langmuir isotherm model and Freundlich isotherm model

Generally, the Langmuir isotherm model is applied in the homogeneous adsorption and monolayer adsorption, and it can be defined as the following equation [5]:

$$\frac{C_e}{q_e} = \frac{1}{q_m b} + \frac{C_e}{q_m} \qquad \text{(S5)}$$

where C_e (mg/L) is the equilibrium concentration of the absorbate, q_e is the

equilibrium adsorption capacity of the MB adsorbed on the PCL/CB[7] nanofibrous membrane.

In contrast, the Freundlich isotherm model is an empirical equation for heterogeneous system and multilayer adsorption, and it is represented as [5]:

$$\ln q_e = \ln K_F + \frac{1}{n} \ln C_e \qquad \text{(S6)}$$

Where q_e and C_e are the same with those of equation 6, K_F (L·mg^{-1}) is the Freundlich constant and n is the heterogeneity factor.

11. Kinetics parameters for the adsorption of MB on nanofibrous membranes

Table S3: Kinetics parameters for the adsorption of MB on PCL and PCL/CB[7] nanofibrous membranes

Sample	Experimental	pseudo-first-order model			pseudo-second-order model			intraparticle diffusion model		
	q_{exp} (mg·g^{-1})	q_e (mg·g^{-1})	k_1 (min^{-1})	R^2	q_e (mg·g^{-1})	k_2 (g·mg^{-1}·min^{-1})	R^2	k_i (mg·g^{-1}· min$^{1/2}$)	C	R^2
PCL	2.37	2.28	0.0499	0.9880	2.49	0.0291	0.9960	0.09295	0.9089	0.6009
PCL/CB[7] (100:50)	4.53	4.32	0.0319	0.9923	4.84	0.0087	0.9958	0.19695	1.2651	0.7136
PCL/CB[7] (100:100)	6.22	5.93	0.0232	0.9921	6.77	0.0043	0.9971	0.28526	1.3190	0.7918
PCL/CB[7] (100:150)	8.41	8.11	0.0224	0.9918	9.27	0.0030	0.9969	0.39144	1.7365	0.7966
PCL/CB[7] (100:200)	9.70	9.34	0.0206	0.9874	10.72	0.0024	0.9938	0.45337	1.8655	0.8180

12. SEM of PCL/CB[7] nanofibers after adsorption of MB

The morphology of PCL and PCL/CB[7] nanofibers after the absorption experiment are shown in Figure S9. It is found that all nanofibers kept their fibrous shape after the adsorption, however the fiber morphology slightly changed compared with that of Figure 1. From Figure S9a, the diameter of PCL nanofibers increases sharply and physical crosslinking between the fibers occurs due to the swelling of PCL in ethanol during a long time (8 h). As shown in Figure S9b and S9c, PCL/CB[7] (100:50) and PCL/CB[7] (100:100) nanofibers also show similar changes because the content of PCL in the nanofibers is relatively higher. With the increase of CB[7] content, the morphology of PCL/CB[7] (100:150) and PCL/CB[7] (100:200) nanofibers (Figure S9d and S9e) kept more constant and the diameter of the fibers changed little. The fibers kept better the fibrous morphology, which indicates that the nanofibrous membrane have good mechanical properties and can easily be removed from the adsorption tail liquid.

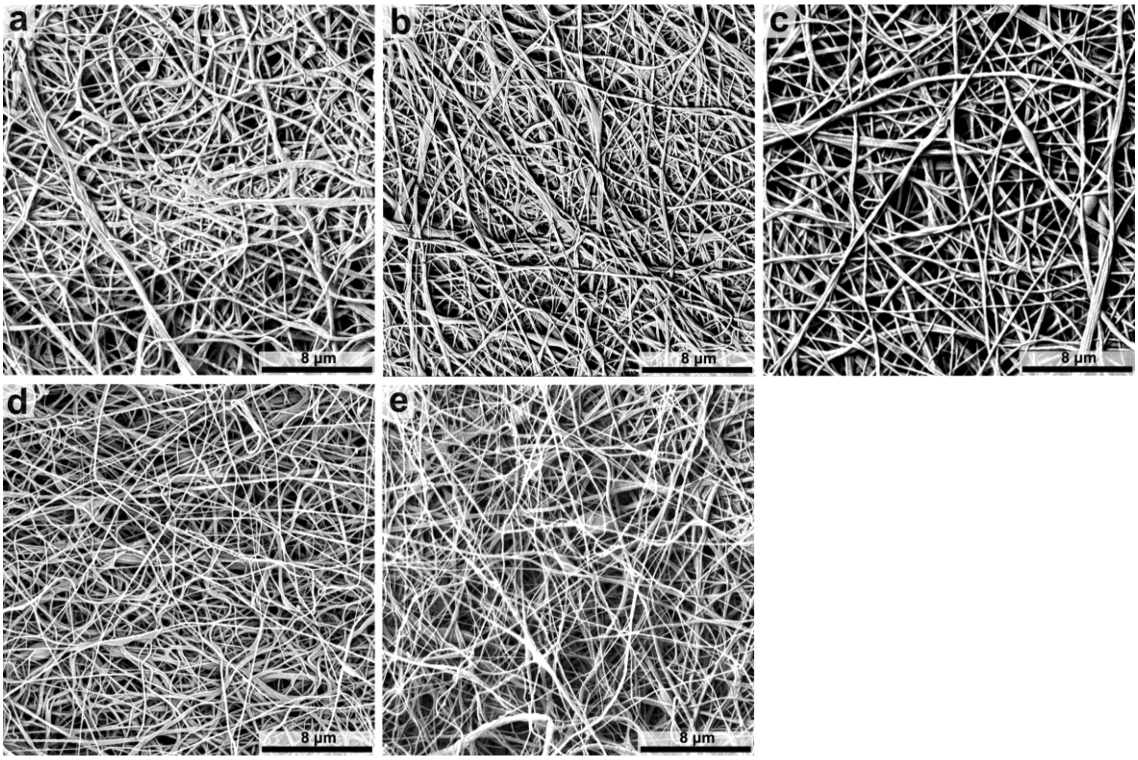

Figure S9: SEM micrographs after adsorption of MB: (a) PCL; (b) PCL/CB[7] (100:50); (c) PCL/CB[7] (100:100); (d) PCL/CB[7] (100:150); (e) PCL/CB[7] (100:200).

References

1. Day, A.; Arnold, A. P.; Blanch, R. J.; Snushall, B. *J. Org. Chem.* **2001**, *66*, 8094-8100.
2. Xie, X.; Li, X.; Luo, H.; Lu, H.; Chen, F.; Li, W. *J. Phys. Chem. B* **2016**, *120*, 4131-4142.
3. Chen, R.; Qiao, H.; Liu, Y.; Dong, Y.; Wang, P.; Zhang, Z.; Jin, T. *Prog. Sustain. Energy* **2015**, *34*, 512-519.
4. Chen, M.; Wang, C.; Fang, W.; Wang, J.; Zhang, W.; Jin, G.; Diao, G. *Langmuir* **2013**, *29*, 11858-11867.
5. Zhao, R.; Wang, Y.; Li, X.; Sun, B.; Jiang, Z.; Wang, C. *Colloid. Surface. B* **2015**, *136*, 375-382.
